# Small Fish, Big Science

**DOI:** 10.1371/journal.pbio.0020148

**Published:** 2004-05-11

**Authors:** Jane Bradbury

## Abstract

The European Union recently awarded 12 million Euros to the ZF-MODELS research consortium to study zebrafish models for human development and disease

Francis Hamilton, the Briton who first described zebrafish (Danio rerio) in 1822, would be astounded to see the scientific attention now afforded to this two-inch-long native of Indian rivers. A fish with no economic worth was how he described this little creature. Yet recently, the European Union awarded 12 million Euros to the ZF-MODELS research consortium to study zebrafish models for human development and disease. When and why did zebrafish swim from home aquaria into research labs, and what can we learn about our biology from this surprising source?

## The Early Days

It was the late 1960s when phage geneticist George Streisinger began to look for a model system in which to study the genetic basis of vertebrate neural development. His passion for tropical fish led him to the humble zebrafish. He was a ‘visionary’, remembers neurobiologist Judith Eisen (University of Oregon, Eugene, Oregon, United States), ‘who laid the groundwork for the use of zebrafish as a developmental model’.

Eisen, who now heads her own research group, went to Oregon in 1983 to work on Xenopus neural development but soon became attracted to zebrafish as a model organism. By the early 1980s, she explains, Streisinger had worked out many of the genetic tricks needed to tackle zebrafish development. What's more, the fish had ‘wonderful embryology’. The embryo, which develops outside its mother, is transparent. ‘You can see different cell types, watch individual cells develop, do transplantation experiments’, Eisen enthuses, ‘and development is quick but not too quick’. Being able to watch individual neurons developing in real time opened up whole new avenues of research for Eisen and other neurobiologists.

## Fast Forward to the Big Screen

**Figure 1:**
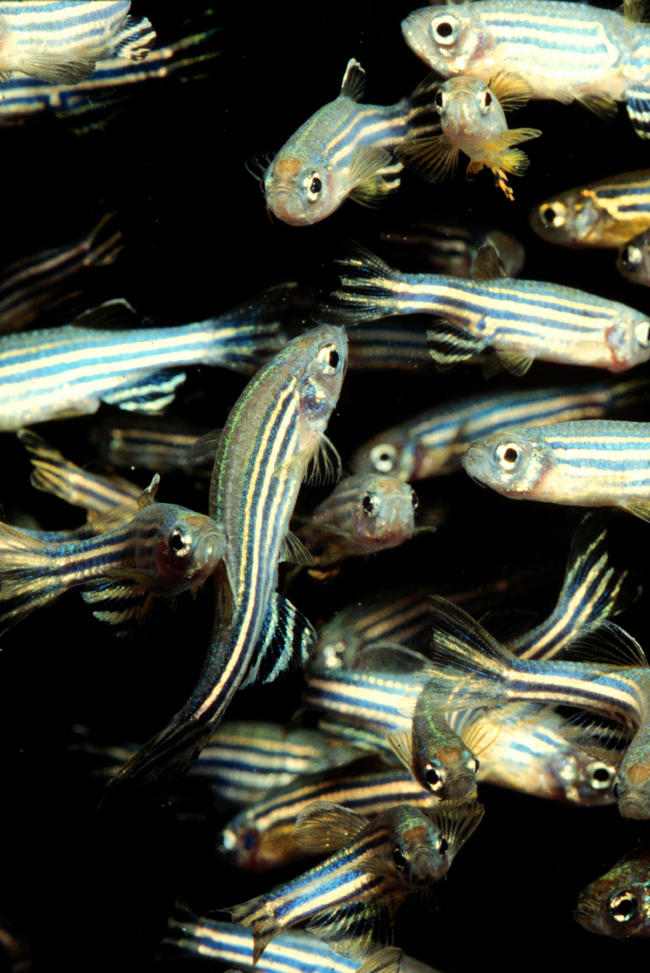
Adult Zebrafish (Image courtesy of Lukas Roth, University College London, London, United Kingdom.)

The properties of zebrafish that attracted Eisen soon attracted people interested in other aspects of vertebrate development to the stripy tiddler (Figure 1). As Eisen comments: ‘No other developmental model has risen to prominence so quickly’. These days more than 3,000 researchers are listed on ZFIN, a United States–based information resource for the zebrafish research community.

The speedy expansion was driven in great part by two genetic screens initiated in 1992–1993 by Christiane Nüsslein-Volhard in Tübingen, Germany, and Wolfgang Driever and Mark Fishman in Boston, Massachusetts. The aim of both screens was to identify genes with unique and essential functions in zebrafish development, and in 1996 an issue of the journal *Development* was dedicated to the mutants that had been isolated and characterised. These screens, says Ralf Dahm (Max Planck Institute for Developmental Biology, Tübingen, Germany), project manager of the ZF-MODELS consortium, ‘were the first major zebrafish projects, and they showed that zebrafish was a model organism to be reckoned with’.

‘That was a fantastic time’, says Derek Stemple, then a postdoc with Driever but now a group leader at the Wellcome Trust Sanger Institute (Cambridge, United Kingdom) and a ZF-MODELS participant. ‘From Wolfgang's lab, I was able to take the mutations that affected notochord development, and have been studying them ever since’. The notochord is an embryonic structure that forms the primitive axial skeleton of the developing embryo, and because mutations affecting notochord development result in shortened embryos, seven of the affected genes have been named after the dwarves in Snow White—zebrafish, like some other developmental models, have many imaginatively named mutants. Stemple now knows the identity of six of these mutated genes, all of which lead to disruption of basement membrane around the notochord.

**Figure 2:**
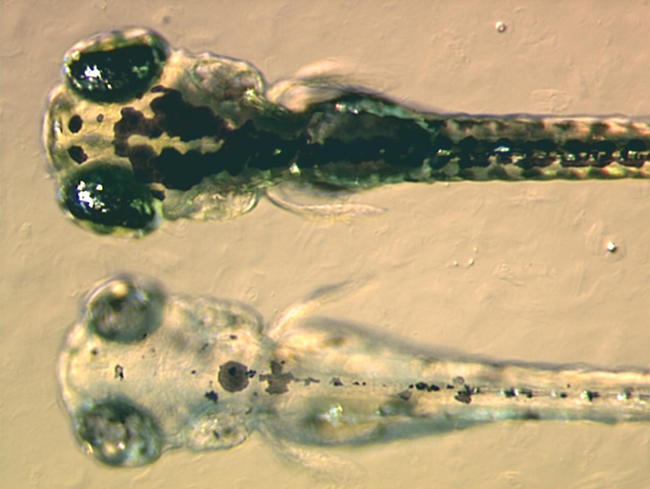
A Zebrafish Pigment Mutant The mutant called bleached blond was produced by insertional mutagenesis. The embryos in the picture are four days old. At the top is a wild-type embryo, below is the mutant. The mutant lacks black pigment in the melanocytes because it fails to synthesise melanin properly. (Image courtesy of Adam Amsterdam, Massachusetts Institute of Technology, Boston, Massachusetts, United States.)

Many mutants from those first two screens are still used by developmental biologists, but another set of mutants has recently been isolated by Nancy Hopkins, Amgen Professor of Biology at the Massachusetts Institute of Technology (Cambridge, Massachusetts, United States). About ten years ago, Hopkins started to develop insertional mutagenesis in zebrafish (Figure 2). In this approach, mutations are caused by the random insertion of viral DNA throughout the fish genome. The inserted DNA acts as a tag, making cloning of mutated genes very straightforward, although the efficiency of the initial insertional mutagenesis is much lower than that of the chemical mutagenesis used in the 1992–1993 screens. Hopkins has isolated 550 mutants in her screen, representing around 400 different genes, and has cloned more than 300 of these genes to date. Some of the fruits of this project are published in this issue of PLoS Biology. Hopkins's group is now collaborating with 25 external laboratories on the annotation of the mutant collection with funding from the National Center for Research Resources, part of the United States National Institutes of Health.

The Tübingen researchers did another chemical mutagenesis screen between 2000 and 2001, and are now starting a third screen of 6,000 genomes as part of the ZF-MODELS project. ‘Each of our screens has built on the previous one by including more specific assays’, explains Dahm. Mutagenesis for the third screen is underway, but the assays, which include looking for defects that specifically affect adults, are still at the pilot stage; this autumn, the project's executive committee, which is headed by Nüsslein-Volhard, will decide which assays to use in the full-scale screen. ‘Just over half the 17 partners in the consortium will come to Tübingen to do screens’, predicts Dahm. ‘By bringing in expertise in different systems in this way we should greatly increase the efficiency of the screen’.

## What Else Will ZF-MODELS Do?

The ZF-MODELS consortium, which is funded under the European Union's Sixth Framework Programme, aims to establish zebrafish models for human diseases, discover genes that will lead to the identification of new drug targets, and gain fundamental insights into human development. ‘We will mainly focus on using advanced technologies that have recently become available’, says scientific coordinator Robert Geisler (Max Planck Institute for Developmental Biology). For example, Geisler's lab will use DNA chip technology to investigate gene expression patterns in zebrafish mutants and so provide increased knowledge of the regulatory pathways that act in zebrafish development.

Consortium members will also use ‘reverse genetics’ to investigate these pathways. In reverse genetics, researchers start with a gene of interest and investigate the phenotypic effect of altering its activity; by contrast, in ‘forward genetics’ the starting point is to look for a particular phenotype and then hunt out the altered gene that is causing it. Two reverse genetics approaches will be used by the consortium. Gene expression will be transiently knocked down with morpholinos, short segments of the gene that block its function. In addition, a recently developed technique known as TILLING (targeting induced local lesions in genomes) will be used to knock out gene activity permanently.

The first step in TILLING is to mutagenise male zebrafish and mate them with untreated female fish, explains Stemple, whose group is one of three ZF-MODELS partners who will use this approach. Offspring are raised to adulthood, and the DNA of each individual is then genotyped for the exon of interest. The consortium already has a collection of 6,000 such individuals, and once a fish carrying a mutation in the gene of interest has been identified, it will be outcrossed to produce offspring, half of which will carry the desired mutation on one of their chromosomes. ‘It is then a matter of identifying these heterozygote fish and incrossing them to get homozygous fish in which you can see the phenotype that correlates with that mutation’, says Stemple.

As well as helping to produce knock-outs for other researchers, Stemple is also using the TILLING technique to develop zebrafish models for muscular dystrophy. Among the genes that are important in notochord development are those that encode laminins. This led Stemple into studying muscular dystrophy because laminins are involved in the human disease. ‘When we used morpholinos to disrupt [the production of] dystroglycan, a laminin receptor, we got a good model for muscular dystrophy’, he explains. Now, he plans to use TILLING to disrupt up to 30 other genes known to be involved in human muscular dystrophy. ‘In particular, we will look for hypomorphic mutants, fish that are viable but on the edge of falling apart’. These mutants can be used to identify small molecules that push the fish into muscular dystrophy. Finding molecules that can cause a disease in this way ‘might give us a handle on something to fix the disease’, says Stemple.

**Figure 3:**
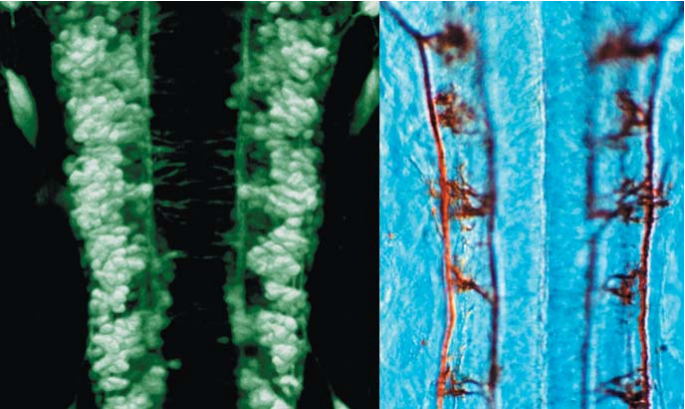
Zebrafish Hindbrain (Left) Dorsal view of GFP-expressing neurons in the hindbrain of a one-day-old zebrafish embryo. (Right) Antibody-labelled axons. (Image courtesy of Dave Lyons, University College London.)

In another strand of the ZF-MODELS project, zebrafish expressing green fluorescent protein (GFP) in specific cells or tissues will be generated and characterised (Figure 3). In such fish, developing structures can be easily imaged over time in the living embryo. One researcher working on this aspect of the project is Stephen Wilson, Professor of Developmental Genetics at University College London (United Kingdom). GFP lines can be made either by attaching to the GFP gene regions of DNA that control, or ‘drive’, GFP expression in selected cell types or by allowing the GFP gene to insert randomly in the genome and looking for fish with specific expression patterns. ‘There are now many lines of fish available with different GFP expression patterns’, says Wilson, ‘and it is important to catalogue their expression so that people can use the most appropriate lines for their research’.

Wilson's own interest is in neuroanatomy. Together with Jon Clarke, another developmental neurobiology group leader at University College London, he plans to analyse GFP lines in which small groups of neurons or particular parts of neurons are labelled, and in this way start to build a detailed reconstruction of early brain neuroanatomy. This, in combination with other work on zebrafish carrying mutations affecting neural development, will give the team ‘a better picture of how a vertebrate brain is built’.

A final, important aspect of the ZF-MODELS project, adds Dahm, is database construction. ‘We will be developing a set of databases that will integrate all of the project data’, he explains. ‘In addition, we hope to integrate our data with that of ZFIN in the United States to make one central zebrafish resource’.

## But Fish Aren't People

The researchers of the ZF-MODELS consortium are understandably excited about participating in what will, says Geisler, bring an already strong European zebrafish community closer together. But zebrafish researchers in the United States are also excited by the ZF-MODELS project. ‘We need big lab models like ZF-MODELS in developmental biology’, says Hopkins, noting that the days of small groups working in isolation are long gone. This consortium, adds Howard Hughes Medical Institute Investigator Leonard Zon (Harvard Medical School, Boston, Massachusetts, United States), ‘will not only have an effect on European zebrafish science but also on how it is done in the United States’.

But how much can zebrafish tell us about human development and disease? A lot, say zebrafish researchers. ‘Fish really are just little people with fins’, says Hopkins. ‘Of course, there are developmental differences between people and fish, and no one pretends that we can answer every question about human development in zebrafish’. Nevertheless, zebrafish studies can provide valuable clues to the genes involved in human diseases and to potential targets for therapeutic interventions. Hopkins provides the following illustration: ‘We have been doing “shelf screens”, in which we go back to our collection of mutants to find all those that affect the development of a single organ. When Zhaoxia Sun, a postdoc in my lab who now has an independent position at Yale Medical School, screened three-day-old fish for cystic kidney disease, she found 12 different genes. Two were known to cause human cystic kidney disease, so we knew we were in the human disease pathway somewhere, but we had no idea what the other genes were’. Hopkins and Sun have since identified the remaining genes, and these point to a single pathway being involved in the human disease.

Developmental geneticist Didier Stainier (University of California, San Francisco, California, United States) is also using zebrafish to study organ development, in particular, heart development. The zebrafish heart is like the early human heart—a tube with an atrium, ventricle, and valves. ‘Everything we have found in the fish is relevant to the human heart’, says Stainier. ‘Obviously, there are additional processes involved in humans, but the basic outline of heart development in fish and people is largely similar’. Stainier has a collection of zebrafish in which valve formation is faulty. ‘Some of the genes we have found will be involved in human congenital valve defects’, he predicts. Knowing the identity of these genes will be useful diagnostically, but, in addition, zebrafish studies can reveal exactly what has gone wrong at a cellular level. The ability to follow individual cells as organs develop is key to this, says Stainier, who reported in March that fibronectin is required for heart development because, by regulating the polarisation of epithelial cells, fibronectin ensures the correct migration of myocardial cells. And in this issue of PLoS Biology, Stainier's lab have identified another zebrafish gene that is involved in heart development—cardiofunk, which encodes a special type of muscle protein.

## A Proliferation of Zebrafish Models of Human Disease

Many researchers are now recognising the value of zebrafish models of human disease. Over the past three to four years, says Zon, this area of research has become a growth industry. The interest in disease models has grown hand-in-hand with the development of morpholinos to knock out specific genes, and the advent of TILLING, says Zon, ‘has set off a whole new fury. There are now large numbers of investigators who will try to knock out their favourite gene and come up with a model’.

**Figure 4:**
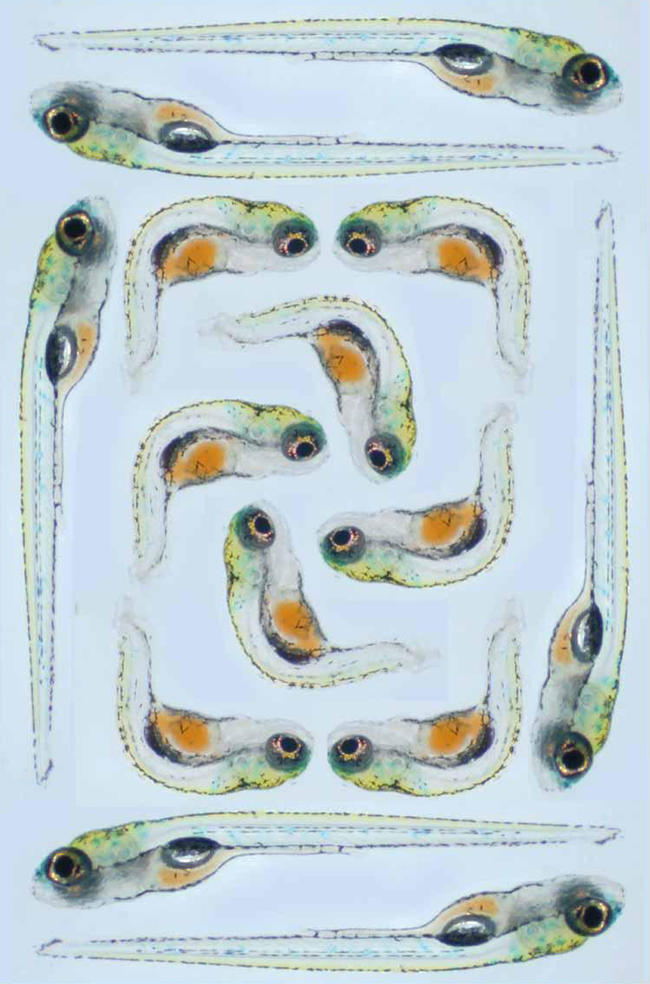
Zebrafish kugelig Mutants The image shows live one-week-old zebrafish embryos. The embryos around the outside are wild-type fish. Those in the middle are a mutant called *kugelig* and have a homozygous mutation in a gene called *cdx4*. Loss of the proper functioning of this gene causes the obvious trunk and tail defects but also causes a reduction in the number of haematopoietic stem cells in the embryos, which therefore become severely anaemic. Studies on this mutant might lead to the discovery of molecules that can drive stem cell differentiation, for example, or could help improve understanding of human haematological malignancies. (Image courtesy of Alan Davidson, Harvard Medical School, Boston, Massachusetts, United States.)

Zon has worked on disease models for blood (Figure 4), blood vessel, and heart disorders but is currently studying zebrafish models of cancer. ‘We started by doing chemical mutagenesis and screened for cell-cycle mutants. These were embryonic lethals, but when we looked at heterozygote carriers of these mutations, some developed cancer at a high rate as adults’. Now Zon and his colleagues have returned to the cell-cycle mutant that yielded this cancer-susceptible heterozygote and are using embryos in high-throughput screening assays to look for small molecules that can suppress the cell-cycle phenotype. These molecules, reasons Zon, may have potential as anticancer drugs.

## And the Future of Zebrafish Research?

Bigger and bigger seems to be the consensus. Chemical screens like Zon's for anticancer drugs can be set up for other human diseases such as muscular dystrophy. Work like Stainier's on organ development may have applications in tissue engineering. ‘If we can find out what drives differentiation in zebrafish’, he suggests, ‘we might be able to do the same for human cells’, making human tissue replacement therapy a practical possibility. And while many zebrafish researchers will continue to study development, others are now moving into the realms of physiology and behavioural studies.

Geisler sums up zebrafish developmental research past, present, and future as follows: ‘No other [vertebrate] organism offers the same combination of transparent and accessible embryos, cost-effective mutagenesis screening, and, more recently, a sequenced genome, [DNA] chip, GFP, and knockout technology’. Add to that the potential of zebrafish embryos as a screening platform for small molecule libraries and the new technologies that allow forward and reverse genetics, and it is clear that zebrafish are not about to revert to being pretty pets swimming in small tanks in the corner of the living room.

## Where to Find Out More

ZF-MODELS

More details of the work included in this European Union Integrated Project can be found at http://www.zf-models.org

ZFIN

The ZFIN Web site, at http://z.n.org/ZFIN, provides an extensive database for the zebrafish community including genetic, genomic, and developmental information; search engines for zebrafish researchers and laboratories; listings of meetings; and links to many other zebrafish sites, including sites with movies of zebrafish development.

The special issue of *Development* (Dec 1; 1996; 123: 1–461) on the first two mutagenesis screens contains 37 research articles and can be freely accessed at http://dev.biologists.org/content/vol123/issue1/index.shtml

## Abbreviations

GFP: green fluorescent protein
TILLING: targeting induced local lesions in genomes
